# Mikrobiom als natürlicher Schutzfaktor: Perspektiven aus der Grundlagenforschung

**DOI:** 10.1007/s00105-021-04831-3

**Published:** 2021-06-11

**Authors:** Thomas C. G. Bosch

**Affiliations:** Zoologisches Institut, Christian-Albrechts-Universität Kiel, Am Botanischen Garten 1–9, 24118 Kiel, Deutschland

**Keywords:** Mikrobiota, Symbiose, Lifestyle-Erkrankungen, Antibiotika, Mikroorganismen, Microbiota, Symbiosis, Lifestyle disease, Antibiotics, Microorganisms

## Abstract

**Hintergrund:**

Eine neue Generation von Technologien deckt eine große Zahl von Mikroorganismen auf, die mit der Haut in einer engen und oft funktionellen Beziehung stehen. Störungen dieser Partnerschaft haben erhebliche Konsequenzen. Seit Jahrzehnten schreitet die Verarmung des Mikrobioms im Zuge eines modernen, globalisierten Lebensstils voran. Bei der Aufrechterhaltung der Gesundheit sind neben den genetischen Aspekten auch die auf der Haut und anderen Organen lebenden Mikroben zu berücksichtigen. Alle Epithelien einschließlich der Haut sind mit einer Vielzahl von Mikroben besiedelt.

**Fragestellung:**

Betrachtet wird die Funktion des Mikrobioms in der Haut und anderen Organen.

**Material und Methode:**

Es erfolgt eine Diskussion von Grundlagenarbeiten.

**Ergebnisse:**

Das Mikrobiom der Haut ist für die Aufrechterhaltung der Gesundheit von großer Bedeutung.

**Schlussfolgerungen:**

Wir brauchen das Verständnis unseres Körpers als multiorganismischer Metaorganismus, um intelligent auf die Herausforderungen einer sich immer rascher ändernden Umwelt reagieren zu können.

## Abstract (en)

**Background:**

A new generation of technologies is uncovering a large number of microorganisms that are closely associated with the skin. Any disturbance of the interaction between skin cells and colonizing microbes has deleterious consequences. The impoverishment of the diversity of microbiome has been progressing for decades as part of a modern, globalized lifestyle. In maintaining good health, the microbes living in and on the skin and other organs must also be taken into account in addition to genetic aspects. All epithelia, including the skin, are colonized with a large number of microbes.

**Objective:**

The function of the microbiome in the skin and other organs is described.

**Materials and methods:**

Basic research papers are discussed.

**Results:**

The microbiome of the skin is very important for maintaining healthy skin.

**Conclusions:**

We need to understand our body as a multiorganismic metaorganism in order to be able to react intelligently to the challenges of a continually changing environment.

Fortschritte in der Mikrobiomforschung haben unsere Denkweise über Gesundheit und Krankheit und unser Bild vom Menschen verändert. Menschen, ihre Organe und ihr Immunsystem funktionieren nur in Zusammenarbeit mit Mikroben. Das Metaorganismuskonzept definiert den Organismus und die mit ihm assoziierten Bakterien als Einheit. Die Störung dieses Zusammenspiels wird als Ursache für viele moderne Krankheitsbilder auch der Haut gesehen.

## Mikroben neu denken

Bakterien besiedeln in großer Zahl und Vielfalt ungefähr 4 Mrd. Jahre länger die Erde als der Mensch [1–3]; 37 % der menschlichen Erbsubstanz können auf bakterielle Vorfahren zurückgeführt werden; sie sind uralter Teil unseres Selbstseins [3]. Weniger als 200 Bakterienarten gelten dabei gemeinhin als ausschließlich krankheitserregend.

Die Hautgesundheit hängt maßgeblich vom Zustand des Hautmikrobioms ab

Bis vor Kurzem wurde den Bakterien auf unserer Haut, in unserer Mundhöhle, in den Atemwegen und im Darm kaum Beachtung geschenkt. Technologische Fortschritte in der Entschlüsselung der Erbinformation haben in den letzten Jahren eine völlig neue und weitgehend unsichtbare Welt sichtbar gemacht und gezeigt, dass sich in unseren Geweben und Organen eine Fülle von genetischen Fußabdrücken entdecken lässt, die unzählig vielen Mikroorganismen zugeordnet werden können. Es ist dadurch möglich geworden, das „Mikrobiom“ einzelner Organe oder des gesamten menschlichen Körpers in einer Vielzahl von kranken und gesunden Zuständen zu bestimmen. Heute wissen wir [4, 5], dass

**Figure 1:**
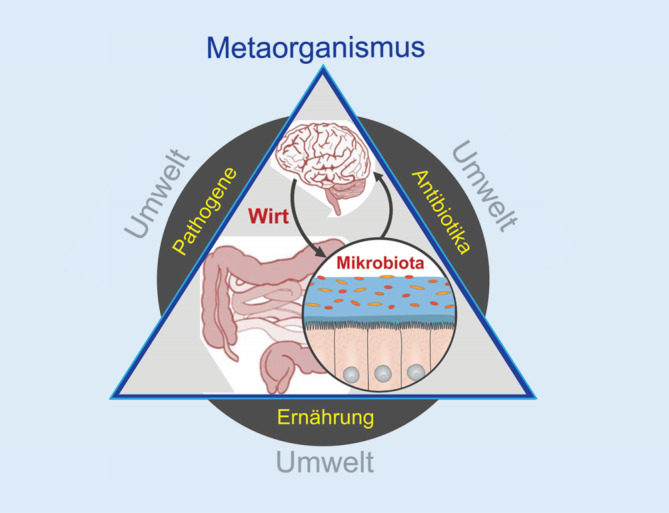

- die allermeisten der uns besiedelnden Mikroben keine Krankheitserreger sind, sondern dass wir sie für unsere Entwicklung und auch zum Schutz vor möglichen infektiösen Erregern brauchen,
- unsere Haut je nach Feuchtigkeit von einigen Hundert und Hunderttausenden von Bakterien pro Quadratzentimeter kolonisiert wird, die bestens angepasst sind, um die auf der Haut nur spärlich verfügbaren Nährstoffe zu nutzen,
- es zwischen unseren Organen wie der Haut, der Mundhöhle, dem Darm und auch dem Gehirn eine enge zelluläre und molekulare Verbindung zu den besiedelnden Mikroben gibt,
- eine Störung der Zusammensetzung des Hautmikrobioms mit einer Reihe von Hauterkrankungen assoziiert ist,
- Organismen immer multiorganismisch sind und es im engeren Sinn keine Individuen gibt, die für sich alleine bestehen können,
- wir nur als Ökosystem existieren in einer evolutionären Partnerschaft mit Mikroben – und wir uns daher besser als Metaorganismus oder Holobiont (Abb. 1) verstehen sollten.

## Was ist ein gesundes Mikrobiom?

Trotz vieler Hinweise auf die Rolle des Mikrobioms für die menschliche Gesundheit ist immer noch unklar, was wir unter einem gesunden oder vorteilhaften Mikrobiom zu verstehen haben. Ein Teil des Problems sind die großen individuellen Unterschiede zwischen den Mikrobiomen scheinbar gesunder Menschen, die sich auf die komplexen Interaktionen von persönlichen Umwelt-, genetischen und Lebensstilfaktoren zurückführen lassen. Relativ kleine Unterschiede können eine unverhältnismäßig große Rolle spielen bei der Frage, ob eine Person relativ gesund ist oder ein erhöhtes Risiko hat zu erkranken. Mit anderen Worten, das gesunde Mikrobiom einer Person ist möglicherweise in einem anderen Kontext nicht gesund. Das Bild wird noch komplexer, weil bestimmte mikrobielle Konsortien sich nicht unbedingt immer aus den gleichen Mikrobenarten zusammensetzen müssen. Aufgrund der erheblichen metabolischen Redundanz sind häufig Gene mit derselben Funktion auf viele Bakterienarten verteilt. Dadurch kann ein gesundes Mikrobiom in unterschiedlichen Individuen durchaus aus unterschiedlichen Arten zusammengesetzt sein. „It´s the song, not the singer …“ [6], auf den es am Ende ankommt.

Dazu kommt, dass sich die taxonomische Zusammensetzung der mikrobiellen Gemeinschaft im Laufe des Lebens verändern kann. Es kommt also nicht auf die einzelnen Arten, sondern auf ihre Funktionalität an. Genauso wie es kein perfektes oder gar „gesundes“ Genom gibt, gibt es auch kein perfektes Mikrobiom. Wenn wir von einem gesunden oder „vorteilhaften“ Mikrobiom sprechen, meinen wir daher mikrobielle Konsortien, die nicht nur mit unserer Lebensweise und unserer ökologischen, soziokulturellen und auch ökonomischen Umwelt harmonieren, sondern auch mit unserem genetischen Hintergrund.

## Mikrobiome sind Filter

**Figure 2:**
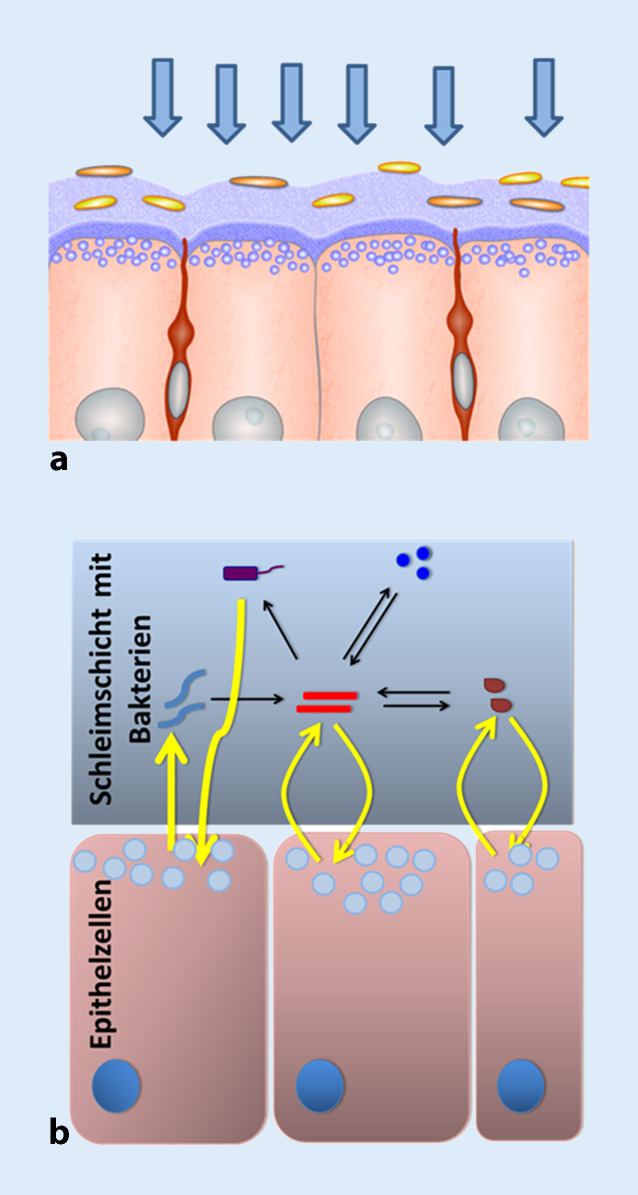

Um zu begreifen, wie der Zusammenbruch dieses Ökosystems der komplexen Lebensgemeinschaft aus Mensch und Mikroben zu Krankheiten führt, müssen wir verstehen, wie die unterschiedlichen Mikroben untereinander und mit unseren Zellen interagieren. Bislang gingen die Zellbiologen davon aus, dass unsere Zellen Signale aus der unmittelbaren Umgebung direkt über ihre Zellwand empfangen und prozessieren. Dafür sind ihre Oberflächen gespickt mit Sensoren und Rezeptoren. Aus dem oben Gesagten geht nun hervor, dass die meisten Epithelien und natürlich auch unsere Hautoberfläche von einer komplexen Gemeinschaft von Bakterien besiedelt sind (Abb. 2). Viele der Signale, die aus der Umwelt ankommen, mag es sich um UV-Strahlen, Nahrung, Temperatur, Bruchstücke und Moleküle von anderen Zellen und vieles andere mehr handeln, werden daher zunächst durch das Mikrobiom gefiltert, bevor sie die Außenmembran unserer Zellen erreichen. Das Mikrobiom dient damit als lebender und schützender Filter zwischen unseren Epithelien und der Umwelt.

## Prinzip Kolonisierungsresistenz

Schon in den 1950er-Jahren wurde im Tiermodell ein Zusammenhang zwischen Dysbiose infolge einer Antibiose und einer deutlich erhöhten Suszeptibilität gegenüber bakteriellen Infektionen beschrieben [7, 8]. Vergleichende Mikrobiomanalysen von Patienten nach Antibiotikabehandlung lieferten erste Hinweise, dass der durch die Einnahme von Antibiotika verursachte mikrobielle Diversitätsverlust mit einem erhöhten Infektionsrisiko einhergeht [9]. Diese Schutzfunktion wird auch als Kolonisierungsresistenz bezeichnet. Symbiotische Mikroben können eine solche Kolonisationsresistenz durch ganz unterschiedliche Mechanismen herbeiführen. Sie können beispielsweise „passiv“ um gemeinsame Nahrungsquellen und ähnliche ökologische Nischen kompetieren. Viele kutane Mikroorganismen können antimikrobielle Moleküle produzieren, die das Eindringen von fremden Mikroben aus der Umgebung verhindern und die Besiedlung hemmen [10–12]. So ist seit Langem bekannt, dass eine Untergruppe von *Staphylococcus*-*epidermidis*-Stämmen die Bildung von *Staphylococcus-aureus*-Biofilmen hemmt [13, 14]. Dies ist von Interesse, weil *Staphylococcus aureus* als ein signifikanter Risikofaktor für eine nachfolgende Infektion gilt [15, 16]. Von *Staphylococcus lugdunensis* ist bekannt, dass es das Wachstum von *Staphylococcus aureus* durch die Produktion des Antibiotikums Lugdunin hemmt [17, 18]. Ein weiteres Beispiel für ein Bakterium, das zur Kolonisierungsresistenz und zum Schutz der Haut beiträgt, ist *Corynebacterium accolens*. Dieses Bakterium verändert die lokale Umgebung der Haut, um das Wachstum von *Streptococcus pneumoniae* zu hemmen [19].

Das Mikrobiom spielt bei bakteriellen Infektionen eine zentrale Rolle

Unter den normalen Hautmikroben scheint sich also eine Reihe von Arten zu befinden, die entweder direkt oder über sekretierte Stoffwechselprodukte ein möglicherweise wirksameres Mittel zur Hemmung opportunistischer Krankheitserreger sein können als die herkömmlichen Antibiotika, gegen die Bakterien auch noch Resistenzen entwickeln können. Quasi als „proof of principle“ für das Translationspotenzial dieser Erkenntnis kann die Beobachtung herangezogen werden, dass eine lokale Anwendung von solchen antimikrobiell wirkenden Hautmikroben die Besiedlung von *Staphylococcus aureus* bei Personen mit atopischer Dermatitis verringern kann [20].

## Mikroben sind Teil des Immunsystems

Die mikrobiellen Mitbewohner sind ein integraler Bestandteil unserer Immunabwehr. Wir wissen seit Langem, dass keimfrei gehaltene Tiere äußerst anfällig für Pilzinfektionen sind [21]. Offensichtlich wird mit dem Entfernen der symbiotischen Bakterien auch der Schutzschild gegen Krankheitserreger wie die in der Umwelt ständig vorhandenen Pilzsporen entfernt. Das gilt nicht nur für das Tiermodell, sondern auch für den Menschen. Jeder, bei dem eine Parodontitisbehandlung mit einer oralen Antibiotikatherapie unterstützt wurde, weiß, wie schnell plötzlich ein Pilz aus der Gruppe der Hefen die Mundschleimhaut besiedeln und zu einer Kandidose führen kann.

Das gilt übrigens nicht nur für die Mikroben in der Mundhöhle, sondern auch für die Mikroben in jedem anderen unserer Organe. Mit Blick auf die Haut wird zunehmend klar, dass sich die Mikrobiomzusammensetzung von erkrankten Hautbereichen (z. B. bei atopischer Dermatitis und Psoriasis) deutlich von der gesunder Haut unterscheidet [18, 22]. Der Verlust von Vielfalt im Mikrobiom scheint zu einer neuen Art von Verwundbarkeit zu führen.

## Vom Mikrobiom zu den Lifestyle-Krankheiten

Funktionelle Studien legen nahe, dass Änderungen in der Diversität des Mikrobioms (vornehmlich aber nicht ausschließlich des Darmmikrobioms) eng mit dem Auftreten von sog. Lifestyle-Erkrankungen assoziiert sind und zu deren Entstehen möglicherweise sogar kausal beitragen. Als Lifestyle-Erkrankungen werden Krankheiten bezeichnet, die mit der Art und Weise zusammenhängen, wie wir als Person oder als Gruppen von Menschen leben. Dazu gehören Stoffwechselerkrankungen wie Adipositas und Typ-2-Diabetes, entzündliche Darmerkrankungen, Asthma, Allergien und Immundefekte und auch eine Reihe neurogenerativer Erkrankungen, die derzeit vorher nicht gekannte Inzidenzen erreichen. Bei den meisten dieser Erkrankungen lässt sich eine deutliche Abnahme der bakteriellen Diversität beobachten, die meist auch mit dem Verlust bestimmter metabolischer Funktionen einhergeht. Die Abwesenheit der Bakterien oder auch die Störung der normalen Bakterienzusammensetzung (Dysbiose) scheint daher mitverantwortlich zu sein für das Auftreten dieser komplexen Erkrankungen [23].

Heute wissen wir von einer Fülle von Faktoren, die die mikrobielle Kolonisierung der Haut und anderer Organe mit Mikroben positiv wie negativ beeinflussen [24–26]. Dazu gehören intensivierte Hygienepraktiken ebenso wie der übermäßige Gebrauch von Antibiotika, Alkohol und anderen Drogen sowie geänderte Geburts- und Säuglingsernährungspraktiken und nicht zuletzt die verringerte Vielfalt in der Ernährung [27].

## Die Beeinflussbarkeit des Mikrobioms

Moderne Sequenziermethoden haben eine Fülle von genetisch bedingten Erkrankungen der Haut (Genodermatosen) aufgedeckt [28]. Diese Krankheiten verlaufen häufig schwer, sind aber in der Regel seltene Krankheitsbilder. Die Möglichkeiten einer entsprechenden Manipulation („Editierung“) des menschlichen Genoms sind Gegenstand vieler ethischer Debatten und nicht praxisrelevant. Die gleichen Sequenziertechnologien haben uns gezeigt, dass die meisten Organe von einem stabilen Mikrobiom besiedelt sind und dass die Störung oder Abwesenheit dieses Mikrobioms (Dysbiose) mit einer Reihe von chronisch inflammatorischen Erkrankungen in Zusammenhang gebracht werden kann. Viele dieser Krankheiten sind zu Volkskrankheiten geworden. Zum Beispiel hatten nach Angaben des Kinder- und Jugendgesundheitssurvey 13,2 % der Kinder in Deutschland bereits einmal in ihrem Leben ein atopisches Ekzem (Neurodermitis). Zwei Drittel der Männer (67 %) und die Hälfte der Frauen (53 %) in Deutschland sind übergewichtig; ein Viertel der Erwachsenen gilt gar als stark adipös. Auch wenn es „das“ gesunde Mikrobiom nicht geben mag, sollten wir daran denken, dass unser Lebensstil reichlich Möglichkeiten bietet, das Funktionieren dieser komplexen Lebensgemeinschaft aus Mensch und Mikroben zu unterstützen –oder auch zu beeinträchtigen. Dabei ist die Aufrechterhaltung eines vorteilhaften Mikrobioms keine leichte Aufgabe: Ernährung, Medikamente, Alkohol und Drogen, aber auch körperliche Aktivität, Stress, Schlaf und auch unsere sozialen Interaktionen haben einen Einfluss auf die Zusammensetzung und Funktionalität des Mikrobioms [29]. Wie auch unsere Arbeiten im Kieler Sonderforschungsbereich „Ursprung und Funktionieren von Metaorganismen“ zeigen (https://www.metaorganism-research.com/), sind Mikrobiome eigene Ökosysteme und bergen eine immense genetische Vielfalt und damit auch einen Reichtum an Enzymen und Stoffwechselprodukten, die der Mensch zu seinem Nutzen erschließen kann. Vieles deutet daher darauf hin, dass sich in Zukunft mit der Manipulation und Nutzung des Mikrobioms interessante Möglichkeiten für sehr gezielte therapeutische Eingriffe ergeben werden.

## Das Mikrobiom ist in akuter Gefahr

Zunehmend wird klar, welch elementare Rolle die mikrobiellen Gemeinschaften für die Organismen spielen, die sie beherbergen. Umso bedauerlicher ist es, dass im Zuge unseres globalisierten Lebensstils die Verarmung des Mikrobioms seit Jahrzehnten voranschreitet. Dieser Prozess hat sich in den letzten Jahrzehnten deutlich beschleunigt und vermutlich erheblich zum Anstieg der vielen Lifestyle-Krankheiten (Fettleibigkeit, Asthma, Nahrungsmittelallergien, Herz-Kreislauf-Erkrankungen, chronische Darmentzündungen, Ösophagusreflux und Neurodermitis) beigetragen [23]. Interessanterweise nehmen all diese Krankheiten dramatisch zu, seit Antibiotika immer häufiger zum Einsatz kommen [30].

Auch beim Einsatz von Antibiotika ist ein Umdenken dringend nötig

Durch die antibiotikavermittelte Vernichtung der Mikroben schwindet die Vielfalt unseres Mikrobioms, was bei vielen Menschen die Widerstandsfähigkeit gegenüber Infektionen herabsetzt. Viele Bakterien auf der Haut und im Darm kehren nach einer Antibiotikabehandlung nur langsam oder gar nicht mehr zurück. Sie fehlen fortan als Teil der menschlichen Immunabwehr. Da auch die Schulung der körpereigenen Immunzellen nicht mehr richtig funktioniert, sind Allergien, Asthma, Diabetes oder chronische Darmentzündungen die Folge. Im Tiermodell wurde überzeugend nachgewiesen [30], dass schon ein kurzer, früh in der Entwicklung erfolgender Einsatz von Antibiotika zu einem Verlust der Vielfalt des Mikrobioms führt.

Aus aktuellem Anlass sei angefügt, dass natürlich auch die gegenwärtigen Maßnahmen zur Bekämpfung der Pandemie weitreichende Auswirkungen auf das menschliche Mikrobiom haben. Wir haben an anderer Stelle betont [23], dass die Implementierung von strengen Hygienepraktiken zur Eindämmung der COVID-19-Übertragung jetzt absolut notwendig ist. Wir sollten aber nicht vergessen, dass eine erhöhte Hygiene auch zu mikrobiellen „Kosten“ führen wird, weil die oben beschriebene Funktionalität unseres Mikrobioms dadurch erheblich verringert wird. Die Mikroben, die wir mit uns herumtragen, sind an vielen grundlegenden Prozessen in unserem Körper beteiligt. Diese Organismen interagieren unter anderem mit den Immunzellen in unserer Haut und lehren sie, nur auf ernsthafte Bedrohungen zu reagieren. Im Mittelpunkt aller Bemühungen um Infektionsschutz sollte daher die Sorge um unser Immunsystem stehen und die Erkenntnis, dass ein vielseitiges Mikrobiom ein wesentlicher Teil davon ist.

## Fazit für die Praxis

- Aus unserer evolutionären Perspektive wird deutlich, dass die einzigartig entwickelte Fähigkeit des Menschen, die Umwelt schnell und schneller zu verändern, zunehmend die Funktionalität unseres Mikrobioms außer Kraft setzt. Infolgedessen können von unseren Vorfahren geerbte Interaktionen zwischen den Epithelien und dem Mikrobiom nicht mehr stattfinden, und wir gefährden unsere Widerstandskraft und Gesundheit.
- Nicht der Mensch alleine, sondern das multiorganismische Kollektiv des Holobionten ist der Patient.
- Wir sollten nie vergessen, dass wir alle und schon immer in einer mikrobiellen Welt gelebt haben und dass die Mikroben einen großen Einfluss auf alle Facetten unserer Existenz haben.

## Glossar

*Antimikrobielle Peptide (AMP)*: Proteine mit kleiner Molekülmasse und antimikrobieller Breitbandaktivität. Diese Peptide sind normalerweise positiv geladen und haben sowohl eine hydrophobe als auch eine hydrophile Seite, die es dem Molekül ermöglicht, in wässriger Umgebung löslich zu sein, aber auch in lipidreiche Membranen einzutreten.
*Dysbiose*: Ein veränderter Zustand (oder ein Ungleichgewicht) der Mikrobiota, der mit einer Änderung der Artenzusammensetzung, Häufigkeit und/oder räumlichen Verteilung verbunden ist und typischerweise mit einer Krankheit verbunden ist.
*Holobiont*: Der Wirtsorganismus und all seine symbiotischen und stabil assoziierten Mikroorganismen. Während der Begriff „Metaorganismus“ eine übergeordnete Einheit definiert, die auf alle Arten von voneinander abhängigen Assoziationen anwendbar ist, ist der Begriff „Holobiont“ auf bestimmte taxonomische Gruppen beschränkt.
*Keimfrei*: Wirtsorganismen, die frei von anderen lebenden Keimen oder Mikroorganismen sind.
*Metaorganismus*: Eine multiorganismische Assoziation, die aus einem makroskopischen Wirt und verschiedenen Mikroorganismen einschließlich Bakterien, Archaea, Pilzen, Viren besteht.
*Mikroben*: Mikroskopisch kleine Organismen, zu denen Bakterien, Archaebakterien, Pilze und Viren gehören.
*Mikrobiom*: Die Gesamtheit der Mikroorganismen und ihres kollektiven genetischen Materials, das in oder auf dem Körper eines makroskopischen Wirtsorganismus vorhanden ist. Häufig als Synonym zu Mikrobiota verwendet.
*Mikrobiota*: Gesamtheit der Mikroorganismen, die in den verschiedenen Bereichen unseres Körpers leben und als „Barriere“ zur Außenwelt dienen, von der Haut zum Darm bis zu den Atemwegen.
*Symbiotische Interaktionen*: Eine enge und normalerweise obligatorische Verbindung zwischen 2 oder mehreren verschiedenen Organismen verschiedener Arten, die zusammenleben, oft – aber nicht notwendigerweise – zum gegenseitigen Nutzen.
